# “Not” in the brain and behavior

**DOI:** 10.1371/journal.pbio.3002656

**Published:** 2024-05-31

**Authors:** Cas W. Coopmans, Anna Mai, Andrea E. Martin

**Affiliations:** 1 Donders Centre for Cognitive Neuroimaging, Donders Institute, Radboud University, Nijmegen, the Netherlands; 2 Max Planck Institute for Psycholinguistics, Nijmegen, the Netherlands

## Abstract

All languages make use of negation, but the neural bases of our cognitive ability to process negation remain unknown. This Primer explores a new PLOS Biology study which shows that humans first interpret negated adjectives as affirmative and later modify their interpretation towards, but never exactly as, the opposite meaning.

## Teaser

Negation is key for cognition but has no physical basis, raising questions about its neural origins. A new study in *PLOS Biology* on the negation of scalar adjectives shows that negation acts in part by altering the response to the adjective it negates.

Language fundamentally abstracts from what is observable in the environment, and it does so often in ways that are difficult to see without careful analysis. Consider a child annoying their sibling by holding their finger very close to the sibling’s arm. If asked what they were doing, the child would likely say, “I’m not touching them.” Here, the distinction between the physical environment and the abstraction of negation is thrown into relief. Although “not touching” is consistent with the situation, “not touching” is not literally what one observes because an absence is definitionally something that is not there. The sibling’s annoyance speaks to the actual situation: A finger is very close to their arm. This kind of scenario illustrates how natural language negation is truly a product of the human brain, abstracting away from physical conditions in the world [1].

The complexity of this abstraction is easy to take for granted. Language structures how this abstraction is performed, and, for that reason, is likely key to understanding how the human brain makes sense of negation [2]. All languages make use of negation [3], suggesting that it is a fundamental property of human cognition. However, the neural bases of our cognitive ability to process negation remain unknown. This is where the structure of language becomes a great advantage to the investigation of negation in the brain. In their new study in *PLOS Biology*, Zuanazzi and colleagues [4] laudably take on this topic in the realm of neurobiology.

The significance of negation in natural language can be seen in how it differs from negation in logic [2] and how different types of linguistic negation affect meaning in different ways. For example, in sentential negation, negation affects the interpretation of the word “or,” which is not true of negation in logic. In the English sentence “The art critic collects work by Sun Yuan or Peng Yu,” the word “or” is interpreted as exclusive-or (XOR), meaning not both. However, when “or” is within the scope of negation (indicated here by square brackets), it is interpreted as inclusive-or (OR), meaning neither. Thus, the sentence “The art critic does not [collect work by Sun Yuan or Peng Yu]” means that the art critic collects work by neither Sun Yuan nor Peng Yu [5].

However, the mere presence of negation is not sufficient to affect the meaning of “or”; “or” must fall within a specific location of the sentence’s syntactic structure relative to the position of “not” in order to be interpreted as inclusive-or. This is why “or” in “The art critic who does not [like collaborative art] collects work by Sun Yuan or Peng Yu” functions as exclusive-or and not inclusive-or: “or” is not in the scope of “not.” The effect of negation on meaning—here, on the meaning of the word “or”—is mediated by hierarchical syntax.

In sentential negation, negation has a categorical effect on the truth conditions of the sentence [2,3,6]. The effect of negation on scalar adjectives works differently. From a logic perspective, if “good” and “bad” are opposites, we might expect “not good” to have the same meaning as “bad.” However, in natural languages, this is generally not true [6]. Instead, the meaning of “not good” tends to fall somewhere on a gradient semantic axis between the meaning of “good” and “bad,” typically closer to “bad” than “good” [7]. In this way, negation highlights an important, structured yet noncompositional interaction between lexical semantics and phrasal syntax.

For these reasons, negation functions as a lens through which Zuanazzi and colleagues are able to observe the impact of abstract, unembodied syntactic structure on meaning, both behaviorally and in the brain [4]. Their behavioral experiments highlight how complex this task is. Negation with “not” presents a cognitive challenge that intensification with “really” does not: Reaction times to negated adjectives are longer, and participants briefly waver over which side of the scale to move their mouse towards. Ultimately, participants’ behavioral results affirm the standard semantics of scalar adjective negation, showing that “not good” is not quite the same as “bad” [6–8]. However, the differences observed in reaction times and mouse trajectories underscore how multifarious the origins of neural differences in scalar adjective processing may be, and, therefore, how challenging the task of analyzing their neural data is.

Faced with this complexity, the study by Zuanazzi and colleagues reveals a nuanced picture: Using a decoding approach, they find that the neural response to “good” when preceded by “not” is more similar to the neural response to “good” preceded by “really” than it is to “bad” preceded by “really.” In other words, the response to “good” is relatively consistent across contexts. This would be expected from a purely feed-forward perspective of language and would, in principle, be derivable from the sensory similarity of different tokens of “good.” What is interesting then is that there is, nevertheless, a difference in the neural response to “good” depending on whether it is preceded by “not.” This shows that negation has a real-time impact on the representation of scalar adjectives, pointing towards the relevance of abstract, unembodied structure-building to negation processing. Though it will take further work to determine whether this difference is what makes “not good” mean something different than “bad,” Zuanazzi and colleagues provide tantalizing progress towards answering this question.

Future research on the neurobiology of negation will undoubtedly build on the results of Zuanazzi and colleagues’ study and expand upon their analytical approach and stimuli. Zuanazzi and colleagues’ decoding approach is best suited for their comparisons of interest: scalar adjectives with uncontested opposites. However, other words and structures present challenges for this approach, raising questions such as what adjective a phrase like “not luminous” ought to be compared to, or what decoder label should be assigned to a phrase like “not touch.” Methodological approaches that can capitalize on the continuity of semantic spaces hold great promise for addressing these phenomena.

Moreover, to understand how the brain negates the meanings of nouns, verbs, or sentences, and whether its mechanisms are similar to those underlying negated adjectives, other types of data will also be invaluable [9]. In particular, data from a wider sample of languages gathered in naturalistic task settings will be instrumental to understanding the neural underpinnings of negation, because negation is structured differently in different languages [3] and its interpretation can be sensitive to pragmatic in addition to syntactic and semantic factors [2–8]. Together, new approaches and datasets following from the study of Zuanazzi and colleagues paint a bright picture for the future of understanding “not” in the brain and behavior.
